# Prospective Memory Function in Late Adulthood: Affect at Encoding and Resource Allocation Costs

**DOI:** 10.1371/journal.pone.0125124

**Published:** 2015-04-20

**Authors:** Julie D. Henry, Sebastian Joeffry, Gill Terrett, Nicola Ballhausen, Matthias Kliegel, Peter G. Rendell

**Affiliations:** 1 School of Psychology, University of Queensland, Brisbane, Australia; 2 School of Psychology, Australian Catholic University, Melbourne, Australia; 3 University of Geneva, Geneva, Switzerland; University College London, UNITED KINGDOM

## Abstract

Some studies have found that prospective memory (PM) cues which are emotionally valenced influence age effects in prospective remembering, but it remains unclear whether this effect reflects the operation of processes implemented at encoding or retrieval. In addition, none of the prior ageing studies of valence on PM function have examined potential costs of engaging in different valence conditions, or resource allocation trade-offs between the PM and the ongoing task. In the present study, younger, young-old and old-old adults completed a PM task in which the valence of the cues varied systematically (positive, negative or neutral) at encoding, but was kept constant (neutral) at retrieval. The results indicated that PM accuracy did not vary as a function of affect at encoding, and that this effect did not interact with age group. There was also no main or interaction effect of valence on PM reaction time in PM cue trials, indicating that valence costs across the three encoding conditions were equivalent. Old-old adults’ PM accuracy was reduced relative to both young-old and younger adults. Prospective remembering incurred dual-task costs for all three groups. Analyses of reaction time data suggested that for both young-old and old-old, these costs were greater, implying differential resource allocation cost trade-offs. However, when reaction time data were expressed as a proportional change that adjusted for the general slowing of the older adults, costs did not differ as a function of group.

## Introduction

Prospective memory (PM) describes the process of remembering to execute previously formed intentions, and is essential for independent living. Laboratory studies indicate that there are moderate age-related declines in PM function [1], with older adults performing more poorly relative to their younger counterparts. However, the magnitude of age-associated decline has been shown to vary systematically as a function of PM and ongoing task parameters. One potentially important parameter is the emotional valence of the PM cue. Emotionally valenced information is preferentially attended to and remembered across all stages of the adult lifespan, but some studies suggest that the magnitude of this effect interacts with age, whereby there is an age-related ‘positivity effect’ [2–3].

To date, three studies have assessed how the emotional valence of the PM cue affects age differences in PM [4–6]. All three of these studies show that emotional valence is beneficial to PM, but differ with respect to the nature of this effect. Specifically, Altgassen et al. [4] found that age differences in PM were eliminated when cues of either positive or negative valence were presented. Rendell et al. [5] found that positive (but not negative) cues enhanced PM function, and that while this enhancement effect was greatest for older adults, age deficits still remained. Schnitzspahn et al., [6] showed that age differences persisted, but were smaller, in response to cues of either valence. Suggested mechanisms for these age-related emotional enhancement effects include increasing the salience of the PM cue [4–5], and enhancing retrospective memory of the cues [6]. However, these prior studies failed to clearly separate encoding and retrieval processes, and consequently could not address whether the effects identified reflected the operation of processes initiated at encoding, retrieval, or a combination of the two.

Encoding refers to the perceptual and cognitive processes which facilitate the conversion of information into a construct for storage. Baddeley’s [7] working memory model provides a theoretical account of cognitive processes involved in the encoding of information and is directly related to long term memory storage. In this model, working memory involves four components; (i) the central executive system, a supervisory system responsible for the coordination of three slave systems, (ii) the phonological loop, a slave system for processing verbal information, (iii) the visuo-spatial sketchpad, a slave system for processing visuo-spatial information and (iv) an episodic buffer, a slave system that is able to integrate information from different slave systems. In addition to modality specific encoding manipulations such as those involving the visuospatial sketchpad or the phonological loop, encoding manipulations that impose demands on multiple components of this model have been shown to be important determinants of retrospective memory performance. These include processing depth [8], mnemonics [9], chunking [10] and of relevance to the present study, emotional valence [11].

Thus, evidence consistently indicates that it is possible to manipulate encoding parameters (including emotional valence) to enhance *retrospective* memory function, while in a separate literature there is evidence that, in some circumstances at least, emotional (i.e., positively or negatively valenced) task parameters might attenuate age related declines in PM functioning relative to neutral task parameters. Integrating these two literatures has potentially exciting implications for the development of interventions for older adults that experience difficulties with PM. This is because, ecologically, there is greater control of how information is presented at encoding. For example, in the situation of providing directions for medication use; physicians are able to influence how this information is presented and the associated valence. Indeed, the importance of encoding is now well accepted in clinical settings, as shown by the many memory rehabilitation interventions that are directed at encoding, such as errorless learning, depth of processing, mnemonics, vanishing cues, chunking, mind mapping and visual imagery.

Consequently, the first goal of the present study was to assess whether manipulating emotional valence at the encoding stage of information processing is an important determinant of PM performance, and if so, whether the magnitude of this effect interacts with age. In service of this goal, we will assess valence effects on PM task accuracy, as well as on costs as operationalized by PM reaction time on PM cue trials. Moreover, although most previous studies on ageing and PM have grouped older adults into a single, homogeneous group, the broader gerontological literature indicates that older adults (relative to their younger counterparts) show greater inter-individual variability, and can be meaningfully separated into young-old and old-old categories. Differentiation of sub-groups of older adults therefore provides an important, but often ignored, opportunity to identify nuances in ageing and prospective remembering. Accordingly, we will investigate how manipulating valence at encoding affects PM performance for young, young-old and old-old adults.

The second goal was to examine the costs of the PM task to the ongoing task, and consequently assess whether any influence of age on PM task performance occurs via relatively automatic or more controlled processes. This is an important and currently unresolved issue, as reflected in two of the most prominent competing explanatory frameworks for event-based PM: The Multiprocess Framework [12] and the Preparatory Attentional and Memory processes (PAM) model [13]. Whereas the PAM asserts that cognitive resources are invariably expended in prospective remembering, with the ongoing and PM task competing for limited cognitive resources, the Multiprocess Framework proposes a dual route model for PM incorporating (i) a controlled route, where prospective remembering requires cognitive resources, similar to the PAM model, but additionally (ii) an automatic route whereby execution of the prospective intent does not impact on the ongoing task.

Because PM paradigms are dual-task in nature, analyses of costs helps determine whether the prospective remembering has been facilitated through automatic or strategic mechanisms. If the PM task was facilitated through an automatic and obligatory system, then it would be expected that the PM processes would not influence ongoing task performance. Alternatively, if PM performance was facilitated through controlled processes, then the allocation of cognitive resources to the PM task would come at a cost to the ongoing task. In the present study, analyses of costs will be used to establish whether the PM phase (i.e., PM and ongoing task) impaired ongoing task accuracy when compared to the no PM (control) condition (i.e., ongoing task only).

## Method

### Ethics statement

This research was approved by the University of Queensland Human Research Ethics Committee. All participants provided their written informed consent prior to taking part in our research.

### Participants

Forty-two young (18–23 years, 40% male), 38 young-old (65–74 years, 63% male), and 29 old-old (75–92 years, 61% male) adults participated. The young adults were students completing the study in exchange for course credit. Older adults were recruited from metropolitan areas and reimbursed $20 AUD. All had normal mental status, as indexed by scores > 82 on the Addenbrooke’s Cognitive Examination-Revised. All participants completed background measures of cognitive function (National Adult Reading Test (NART), Verbal fluency and the Trails (Parts A and B) as well as a measure of wellbeing (Satisfaction With Life Scale). Descriptive and inferential statistics for these measures are reported in Table 1. It can be seen that the only group differences to emerge were on the Trails Part A and the NART. These effects reflected slower psychomotor speed for both young-old and old-old relative to young adults but higher crystallized intelligence for both young-old and old-old relative to young adults (all *p*s <.05), respectively.

**Table 1:** Background characteristics of the three groups (younger, young-old and old-old adults).

Characteristic	Young		Young-old		Old-old		Inferential statistics		
	*M*	*SD*	*M*	*SD*	*M*	*SD*	*F*	*p*	η_p_^2^
*Demographics*									
Education	13.26	1.45	13.39	3.25	11.55	3.95	3.90	.02	.069
*Wellbeing*									
SWLS	23.43	6.67	26.16	5.98	25.48	5.84	2.77	.07	.050
*Cognition*									
NART FSIQ	110.33	5.23	114.16	6.68	114.90	6.86	4.90	.01	.092
Verbal fluency	44.88	17.83	47.45	13.59	45.03	12.89	0.34	.72	.006
Trails A	21.55	5.25	32.29	10.48	36.24	7.88	32.57	<.01	.381
Trails ratio	1.71	1.27	1.67	0.99	1.58	1.05	0.11	.90	.002

*Note*: SWLS refers to the Satisfaction With Life Scale; NART FSIQ refers to the National Adult Reading Test Full Scale Intellectual Quotient.

## Materials and Design

A 3 x 3 x 2 mixed factorial design was used to investigate the effect of valence at encoding on PM performance, as well as any associated resource allocation costs. The three prior PM studies that identified interactions between age-group and valence identified η_p_^2^ values of.04 [6],.05 [4], and.07 [5]. For η^2^, Cohen [14] defines values of.01 as small,.059 as medium &.138 as large, and it may be appropriate to use the same reference values for η_p_^2^ when ANOVAS are not overly complex [15]. Consequently, prior studies point to there being an interaction of moderate magnitude between age-group and valence, and this effect size was used to inform our power analysis. Assuming a moderate-sized effect where α = .05, the power to detect a significant effect of age group across any of the six levels of valence and ongoing task phase that are crossed with age group for PM valence; across any of the six levels of age group and ongoing task phase that are crossed with PM valence; and for ongoing task phase across any of the nine levels of age group and valence that are crossed with ongoing task phase, is >.80.

The between-subjects variable was *age group* (young, young-old, old-old), and the within-subjects variables were *valence* (positive, negative, neutral) and *ongoing task phase* (no PM, PM), where valence referred to the valence of the PM exemplar shown during instructions.

E-prime (version 2.0) was used to develop and present the computer-based task which had (i) an ongoing-only phase (1-back paradigm working memory task), and (ii) a PM phase (counterbalanced). In the 1-back task participants were shown a series of slides with each slide displaying two images. They had to decide whether either of the images on the current slide were the same as any of the images from the slide presented immediately prior to that currently shown (i.e., 1-back).

The 1-back images were 372 IAPS [16] images that were randomly selected from the IAPS corpus after unsuitable images were removed (images that were potentially distressing or inappropriate, or had unusually salient or atypical perceptual features). Of the original 1194 IAPS pictures, 526 were deemed suitable, from which 372 images were randomly selected for use.

For the PM phase, a PM task was embedded into the 1-back paradigm. This involved pressing a specified key when a picture belonging to one of three defined semantic categories appeared (dogs, babies and insects). The pictures of dogs, babies and insects used to illustrate these three semantic categories at encoding were positive, negative or neutral in valence (counterbalanced). The exemplars of babies, dogs and insects shown during the PM task itself (i.e., the PM cues presented during the retrieval phase) were always neutral in valence, and did not include any of the exemplars shown at encoding. Participants were told prior to starting the PM task that the specific exemplars of the semantic categories may differ to those that they had previously seen.

The PM phase consisted of 150 trials with PM targets occurring at every 16^th^ interval. This allowed for nine opportunities to execute the PM intention, with the nine PM targets equally distributed between each of the three semantic categories, i.e., dogs, babies and insects. Overall, the PM phase included 9 PM targets, 73 1-back targets and 68 1-back non-targets. Participants were told to treat both the ongoing and PM task with equal importance.

Specifically, prior to commencing the PM phase, each participant was shown two exemplars of dogs, two exemplars of babies and two exemplars of insects, with these three semantic categories varying systematically in affective valence. PM exemplars were obtained from the IAPS corpus and via an internet search. All images were independently validated for affect and arousal by 33 participants (33% male, mean age 29) using similar instructions to the original IAPS validation study [14]. Thus, participants’ responses were rated on a 9 point scale ranging from “negativex (1) to “positive” (9) to index valence, and “calm” (1) to “excited” (9) to index arousal. Mean affect and arousal ratings for the 18 PM exemplars presented at encoding are reported in Table 2.

**Table 2:** Mean affect and arousal ratings of prospective memory exemplars and cues.

Semantic category	PM exemplar mean	
	Affect	Arousal
*PM Exemplar*		
Negative dogs	2.22	5.70
Negative infants	2.18	5.49
Negative insects	3.18	5.38
Neutral dogs	4.34	5.48
Neutral infants	4.53	5.36
Neutral insects	4.82	5.22
Positive dogs	7.68	4.85
Positive infants	8.12	4.39
Positive insects	6.81	3.37
*PM Cue*		
Dogs	5.31	5.12
Infants	5.00	4.94
Insects	4.65	5.10

*Note*. Participants’ responses were rated on a 9 point scale ranging from “negative” / “calm” (1) to “positive” / “excited” (9), with (5) affectively neutral..

An independent set of neutral images to be used as PM retrieval cues were also identified from the IAPS corpus and via internet searches. Ratings were again obtained from the 33 participants described previously. As shown in Table 2, the mean affect and arousal ratings for the PM cues presented at retrieval were neutral for all three semantic categories.

### Procedure

After completing several of the background measures, participants undertook the computer-based task. This commenced with an example of the ongoing task and two practice rounds. Participants were then given the PM task instructions. Once the experimenter was satisfied that participants understood these instructions, a 15 minute delay interval was introduced in which the remaining measures were completed. After this delay, participants were directed to proceed with the experiment. No prompts were provided for the PM task. At the conclusion of the PM phase, participants were asked to recall the PM task, as a manipulation check. This led to the exclusion of two young-old and six old-old participants.

### Data Analysis

Proportion of correct PM targets was the primary dependent variable of interest. For analyses focused on valence costs, because longer reaction times typically indicate greater effort, reaction time to complete the PM task on the PM cue trials was assessed as a function of valence encoding condition. Reaction time data for correct responses on the ongoing task were also collated for both the ongoing-only and PM phases of the experiment. Consistent with other PM studies [17], a stable measure of ongoing task performance in the PM phase was obtained by excluding (i) the first three trials of the PM phase, (ii) the PM cue trial and every three trials after a PM cue and (iii) outliers as determined by a reaction time exceeding two standard deviations from the participant’s phase mean. For all analyses, where assumptions of sphericity were violated, Greenhouse-Geisser corrected statistics are reported.

## Results

**Fig 1:**
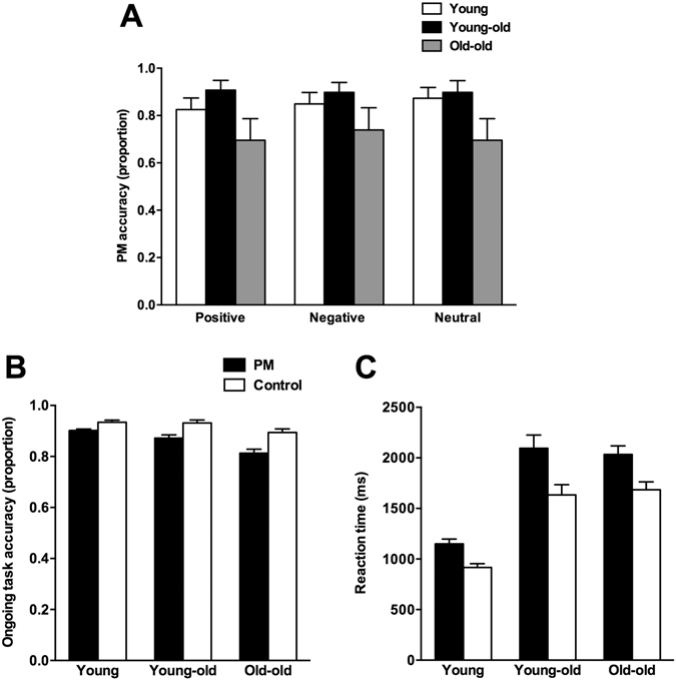
Prospective memory accuracy as a function of emotional valence (Fig 1a); ongoing task accuracy (Fig 1b) and reaction time (Fig 1c) as a function of ongoing task phase (PM, no PM). Bars represent one standard error of the mean.

Fig 1a shows the mean PM accuracy as a function of valence (positive, negative, neutral), and group (young, young-old, old-old). These data were analysed with a 3 x 3 mixed design ANOVA. As can be seen in Fig 1a, there was a main effect of group, *F*(2, 105) = 5.43, *p* = .006, η_p_^2^ = .094, where old-old adults (*M* = .61, *SD* = .47) were outperformed by both young adults (*M* = .87, *SD* = .33, *p* = .010) and young-old adults (*M* = .86, *SD* = .33, *p* = .015). There was no significant effect of valence, *F*(2, 210) = 0.37, *p* = .689, η_p_^2^ = .004, and no significant interaction between the two, *F*(4, 210) = 1.05, *p* = .381, η_p_^2^ = .02.

To assess valence costs, proportional change reaction time scores were analyzed as a function of valence (positive, negative) and group (young, young-old, old-old). Proportional change scores were calculated to adjust for the general slowing of older adults. These scores were calculated as the difference in reaction time for positive (or negative) minus neutral PM trials divided by the reaction time for neutral PM trials. The results revealed that there was no significant main effect of group, *F*(2,92) = 2.29, *p* = .107, η_p_^2^ = .05, no significant main effect of valence, *F*(1, 92) = 0.94, *p* = .334, η_p_^2^ = .01, and no significant interaction between the two, *F*(1,92) = 0.12, *p* = .887, η_p_^2^ <.01.

The next set of analyses focused on whether ongoing task accuracy was compromised when there was the additional requirement to engage in PM monitoring. This was assessed in two different ways—ongoing task accuracy, and ongoing task reaction time.

Ongoing task accuracy was analysed with a 2 x 3 mixed design ANOVA, with the within groups variable of ongoing task phase (no PM, PM), and the between-groups variable of group. As can be seen in Fig 1b, there was a main effect of ongoing task phase, *F*(1, 105) = 19.15, *p* <.001, η_p_^2^ = .15, where performance in the PM phase (*M* = .85, *SD* = .07) was less accurate than in the no PM phase (*M* = .88, *SD* = .08). There was also a main effect of group, *F*(2, 105) = 17.12, *p* <.001, η_p_^2^ = .10, reflecting overall poorer performance for old-old (*M* = 0.82, *SD* = .08) relative to both young-old (*M* = 0.87, *SD* = .09, *p* = .012) and young adults (*M* = 0.91, *SD* = .05, *p* <.001). Young adults were also more accurate than young-old adults (*p* = .008). There was no significant interaction between ongoing task phase and group, *F*(2,105) = 1.60, *p* = .208, η_p_^2^ = .03.

Fig 1c shows the mean ongoing task reaction time as a function of ongoing task phase (PM, no PM) and group (young, young-old, old-old). These data were analysed with a 2 x 3 mixed ANOVA. There was a significant main effect of ongoing task phase, *F*(1, 105) = 112.21, *p* <.001, η_p_^2^ = .52, as well as a significant main effect of group, *F*(2, 105) = 38.32, *p* <.001 η_p_^2^ = .42. There was also a significant interaction between ongoing task phase and group, *F*(2, 105) = 3.62, *p* = .030, η_p_^2^ = .06.

This interaction was analysed with tests of simple effects. The effect of group was significant within each task phase: PM, *F*(2, 105) = 32.61, *p* <.001, η_p_^2^ = .38; no PM, *F*(2, 105) = 38.95, *p* <.001, η_p_^2^ = .43. Tests to follow up these effects revealed that, for the PM phase, younger adults (*M* = 1170.3, *SD* = 311.5) were faster than both young-old (*M* = 2074.8, *SD* = 790.9, *p* <.001) and old-old adults (*M* = 2054.2, *SD* = 473.2, *p* <.001), but the latter two groups did not differ (*p* = .883). For the no PM condition, young adults (*M* = 921.6, *SD* = 244.4) were faster than both young-old (*M* = 1645.1ms, *SD* = 621.9, *p* <.001) and old-old adults (*M* = 1767.0ms, *SD* = 401.6, *p* <.001), but again these latter two groups did not differ (*p* = .277).

Further tests of simple effects revealed that ongoing task phase was significant within the three groups: young adults, *F*(1, 105) = 26.84, *p* <.001, η_p_^2^ = .20, young-old, *F*(1, 105) = 72.49, *p* <.001, η_p_^2^ = .41, and old-old, *F*(1, 105) = 23.84, *p* <.001, η_p_^2^ = .16. In terms of the pattern of these simple effects, young adults were faster in the no PM phase (*M* = 921.6, *SD* = 244.4) relative to the PM phase (*M* = 1170.3, *SD* = 311.5, *p* <.001). This was also true for young-old (*M*s = 1645.1 and 2074.8 ms respectively, *p* <.001), and old-old (*M*s = 1767.0 and 2054.2, respectively, *p* <.001). Taken together, the interaction effect reflects the fact that while the PM phase incurred a cost of increased reaction time, this cost differs as a function of age (Δ between phases = 248.7, 429.7 and 287.1, for young, young-old and old-old respectively).

However, these analyses did not adjust for the general slowing for the older adults. The next step in analyses was therefore to further assess age differences in costs to the ongoing task by analyzing proportional change reaction time scores as a function of group (young, young-old, old-old). These scores were calculated as the difference in reaction time during the PM phase minus reaction times during the no PM phase, divided by the reaction time during the no PM phase. The results revealed that there was no significant main effect of group, *F*(2,105) = 2.44, *p* = .092, η_p_^2^ = .04. Thus, in contrast to the previous analyses (which suggested that costs differed across the three groups), these data instead suggest that there are no differential resource allocation costs as a function of age-group (proportional change = .29 .27 and .18).

Finally, it is worth noting that in the present study PM hit accuracy was defined as correctly responding to the PM target in the trial in which the target appeared. However, because after a participant responded to the ongoing task the next trial appeared, reduced PM could potentially reflect a difficulty inhibiting the ongoing response in time, as opposed to failing to remember the intention *per se*. For this reason, we re-ran the primary analyses, but also allowing ‘little late’ responses to contribute to the accuracy score. Little late was defined as executing the PM action within one and three trials following presentation of the target. The pattern of results was unchanged in these analyses.

## Discussion

Contrary to predictions, the provision of positively or negatively valenced information at encoding did not enhance PM accuracy for any of the three age groups assessed. Moreover, when valence costs were assessed by comparing reaction time on the PM cue trials across the three encoding conditions (positive, negative, neutral), no main effect of valence emerged, nor was there any interaction with age. Taken together, these data imply that, at least in some circumstances, the valence of information presented at encoding may not be an important determinant of PM function. Further, they suggest that this (absence) of effect may be invariant across different stages of the adult lifespan.

Only one prior aging study has attempted to disentangle encoding and retrieval PM processes. However, in Rendell et al.’s [5] study emotion may have still been confounded at retrieval due to the emotional valence associated with the *content* of the PM task. One advantage of the present paradigm was that both the PM *cue* and PM *content* were emotionally neutral. Thus, although null effects always need to be interpreted with some caution since there are many reasons that could lead to a non-significant null hypothesis test other than the true relationship representing no difference, the present data provide some of the strongest evidence to date that manipulating emotional valence at encoding may be neither beneficial nor detrimental to PM functioning.

Further research is now needed to establish whether PM might instead be facilitated through a combination of encoding and retrieval processes, or retrieval processes alone. This is because, as noted previously, emotion does appear to benefit older adults’ PM in at least some circumstances, as reported by the enhancement effect of Altgassen et al. [4] and Schnitzspahn et al. [6], and the positivity enhancement effect by Rendell et al. [5]. The finding of null effects in the present study (but significant effects in these other studies) does not seem easily attributable to sampling error, as the sample sizes were broadly equivalent. One possibility is that specific factors such as the type of stimuli (e.g., visual versus verbal), nature of the experimental paradigm (e.g., Virtual Week, Einstein and McDaniel paradigm), type of PM cue manipulation (e.g., focal versus non-focal), and nature of the ongoing task demands (e.g., high or low in engagement) influence the effects of emotion on PM functioning.

Another important finding to emerge was the main effect of age on PM accuracy, with old-old adults less accurate on PM tasks than both the young-old and younger adults, with these latter two groups not differing. Young-old and old-old groups have been referred to as the third and fourth age, respectively [18–19], and it has been previously argued that young-old adults are more similar to younger than old-old adults in many important characteristics [19]. The present results align with this broader gerontological literature in showing that there are meaningful differences between young-old and old-old in cognitive function, and reinforce the importance of not treating older adults over the age of 65 as a single, homogeneous group.

Indeed, inconsistent PM performance in the context of ageing is well documented [1] [20]. The Multiprocess Framework is often used to explain such variability, with the automatic route used to explain comparable PM performance between younger and older adults, and the strategic route used to explain age related declines. This is because the cognitive processes associated with the strategic route of prospective remembering, such as environmental monitoring for PM cues, rehearsal of prospective intent and interrupting the ongoing activity in order to execute the PM task, have each been shown to decline with age [21–22].

The findings of the present study, showing comparable PM accuracy for young and young-old adults, might therefore be interpreted as evidence for a primary role of automatic processes. Such an explanation seems especially plausible given the use of focal PM cues, which have been shown to reduce age associated decline [23], and the use of visual stimuli, which has been shown to enhance PM performance for both young and old [24]. Indeed, while uncorrected reaction time data suggested that reaction time costs varied as a function of age-groups, adjusting these data for general slowing instead showed that there were no differential resource allocation costs across the three groups.

However, it still seems likely that the PM task in the present study imposed at least some demands on controlled strategic resources. This is based on two findings. Firstly, PM accuracy for old-old adults was significantly lower than for both of the other age-groups. Secondly, cost analyses also showed that PM phase (which included both the PM and the ongoing task) impaired ongoing task accuracy when compared to the no PM condition (i.e., the ongoing task only). These data indicate that, for all three groups, performance on the ongoing task and PM task were competing for the same attentional resources.

Indeed, analysis of costs offers an important method to resolve the wider debate between the Multiprocess Framework and the PAM model. Specifically, cost analyses are central to the falsifiability criterion for each explanatory model. For instance, to reject the Multiprocess Framework reliable evidence needs to demonstrate a PM cost to the ongoing task in a paradigm where the automatic route of prospective remembering would be unequivocally used. In the alternative, Smith, Hunt, Mcvay and McConnell [25] provide their own falsifiability criterion to the Preparatory Attentional and Memory processes theory, stating that their model may be disproved if, “successful performance of a PM task [is] accompanied by convincing evidence of no disruption of a sufficiently sensitive and demanding background task” (p735).

One possible compromise between these two theories is that the dual route model of the Multiprocess framework may in fact represent two poles on a continuum, such that the degree of automaticity varies from being relatively-resource free to highly-resource intensive. This may account for findings which show reduced but not eliminated age-associated differences under certain task parameters [23, 26]. Consistent with such a possibility, Gilbert, Hadjipavlou and Raoelison [27] proposed a computational model of PM in which there is, “a graded continuum between controlled top-down monitoring for PM targets versus pure bottom-up triggering” (p. 11). In their study, in addition to computational modelling, empirical data from two studies was presented that provided support for this theoretical approach.

Finally, several limitations of these data need to be acknowledged. First, in the present study (as with the original IAPS normative dataset), PM-cue exemplars were piloted only on younger adults. A limitation of this approach was that it did not directly establish whether the valence levels of the PM-cue exemplars were comparable for young and older adults. Nevertheless, it is important to note that Grühn and Scheibe [28] found that younger and older adults’ valence ratings of IAPS stimuli were highly correlated, noting that there was, “a general consensus among age groups in evaluating which picture is more negative or more positive” (p. 519). It should also be noted that in the present study PM accuracy levels were quite high, introducing the possibility that age effects may have been masked by near ceiling effects. Since ceiling effects are an acknowledged problem for much of the broader PM and ageing literature, this reinforces the need for additional indices of performance such as reaction time to be used, where ceiling effects are not a possibility.

In conclusion, these data suggest that manipulating emotional valence at encoding is neither beneficial nor detrimental to event-based PM, as reflected both in PM task accuracy, as well as in costs as operationalized by PM reaction time on PM cue trials. This suggests that the emotional effects on PM reported in previous studies reflected retrieval processes alone, or the combination of encoding and retrieval processes and other task parameters. The results also point to an important distinction between young-old and old-old adults, and further highlight the need to take into account resource-allocation costs when interpreting PM performance.

## Supporting Information

S1 Data Supporting data. (SAV)
